# Molecular characterization and phylogenetic analysis of feline hemoplasmas in domestic cats in Iran

**Published:** 2017-03-15

**Authors:** Fereshteh Ghazisaeedi, Nahid Atyabi, Taghi Zahraei Salehi, Iraj Ashrafi Tamai, Saeid Tabatabaei, Solmaz Chegeni

**Affiliations:** 1 *Department of Veterinary Internal Diseases; *; 2 * Department of Microbiology and Immunology, Faculty of Veterinary Medicine, University of Tehran, Tehran, Iran.*

**Keywords:** Anemia, Cat, Feline hemoplasma, Iran

## Abstract

Three known feline hemoplasmas are *Mycoplsama haemofelis*, ‘*Candidatus* *Mycoplasma haemominutum*’ and ‘*Candidatus* *Mycoplasma turicensis’*. They are described as cause of feline infectious anemia in domestic and wild felids. Other blood parasites or blood-related pathogens like concurrent retroviral infections may deteriorate the clinical condition and severity of anemia. The aims of this study were molecular characterization and phylogenetic analysis of hemoplasmas in domestic cats in Iran for the first time. Blood samples were collected from 185 healthy and diseased domestic cats. Blood smears were prepared and hematological parameters were measured to determine possible anemia. Using 16S rRNA gene universal and species specific polymerase chain reactions with the following sequencing, 47 (25.40%) of cats were hemoplasma positive. Also, 17.02%, 72.50% and 40.40% of total positive samples were *M.* *haemofelis*, ‘*Ca*. M. haemominutum’ and ‘*Ca*. M. turicensis’ infected, respectively. 10 (21.20%) of hemoplasma positive cats had anemic blood profiles (HCT < 24.00%). All M. *haemofelis* infected cases were included. Partial 16S rRNA gene phylogenetic analysis revealed a high identity between the hemoplasma species found in this study and domestic cat sequences existing in GenBank. Phylogenetic analysis revealed 94.00% to 100% sequence identity between sequences of this study and existing sequences in Genbank. All hemoplasma isolates in this study were grouped within a single clade and additionally subdivided into two groups; haemofelis group including *M.* *haemofelis* and ‘*Ca*. M. *turicensis’* and haemominutum group including ‘*Ca*. M. haemominutum’.

## Introduction

Haemoplasmas are haemotropic mycoplasma bacteria in a very wide range of mammalians^1^ which are reclassified based on 16S rRNA gene from Rickettsia to the Mycoplasma genus. ^2^ Three feline haemoplasma species are described in domestic cats including *M. haemofelis, *‘*Ca*. M. haemominutum’ and ‘*Ca*. M. turicensis’^1^^-^^4^ causing hemolytic anemia in cats mostly in *M. haemofelis *infected cases.^5^^,^^6^ Infected cats have no specific clinical signs typically in ‘*Ca*.* M. haemominutum’* and ‘*Ca*. M. turicensis’infection.^7^ Co-infection of haemoplasmas with some other pathogens like feline leukemia virus (FeLv) can lead to a severe and life threatening anemia.^8^^,^^9^

Since haemoplasmas could not be cultured^10^^,^^11^ and cytological examinations of blood smears are not reliable, ^12^^,^^13^ other diagnostic methods mainly molecular assays are investigated.^14^^-^^17^ Using molecular techniques like polymerase chain reactions (PCR), detection, quantification and follow up of the treatment in hemotropic mycoplasmas are practicable.^15^^,^^16^ In addition, partial genome sequencing of common 16S rRNA gene in isolates from different hemoplasma species and complete genome sequencing project of *M. haemofelis *and *Ca. Mycoplasma haemominutum*,^18^^-^^20^ facilitate studies about the evolution, pathogenesis and interspecies transmission in haemoplasmas. In a recent study from our group, the first report on the presence and clinical and hematological aspects of feline hemotropic mycoplasmas were described in domestic cats in Iran.^12^

The aim of this study was to investigate feline haemolplasma species in domestic cats with an approach to sequencing and phylogenetic analysis to determine the identity of detected isolates and compare to worldwide cat-derived isolates due to expansion of our knowledge about these hemotropic mycoplasmas. 

## Materials and Methods

**Sample collection. **EDTA-anticoagulated blood samples, collected from femoral vein into 2.5 mL tube (FL Medical S.r.l., Torreglia, Italy), were obtained from 185 healthy and diseased domestic cats (112 males and 73 females) of random ages, referred to three main referral diagnostic centers and an animal shelter between 2012 and 2014 in Tehran, Iran. Hematological parameters including white blood cell count, red blood cell count, hematocrit (HCT), hemoglobin concentration, mean corpuscular volume, mean corpuscular hemoglobin concentration and platelets count were measured using an automatic hemocytometer (Model Hema-screen 18; Hospitex diagnostic, Florence, Italy). Blood smears were prepared due to initial hemoplasma examination. Subsequently, blood samples were subjected to DNA extraction procedure for further molecular investigations.

**DNA extraction. **DNA was prepared of 100 µL blood sample using blood pathogens extraction kit (Molecular Biological System Transfer, Tehran, Iran) following the manufacturer's instructions and stored in – 20 ˚C prior to further investigations. For evaluating the extraction kit specificity and sensitivity, distilled water used as a negative control. The serial dilution of control positive samples (cloned DNA isolated from clinical cases, from the School of Veterinary Sciences, Bristol University, Bristol, UK and Bologna University, Bologna, Italy) with known copy number (down to 50 copy number) was extracted with the kit and subjected to the detecting conventional PCRs of feline haemolplasma species. 

**Diagnostic PCR assays. **The control PCR to amplify a fragment of glyceraldehyde-3-phosphate dehydrogenase gene was applied to determine the quality of PCR procedure.^21^ Screening was performed based on previously described universal haemotropic mycoplasma conventional PCR detection method.^22^ The positive samples with universal hemotropic mycoplasma PCR were subjected to the species specific conventional PCR of three feline haemolplasma species through formerly designed conventional PCR assays.^23^^,^^24^ Data are shown in Table 1. 

**Table 1:** List of primers used in this study

**Species**	**Name**	**Primer sequence**	**Size of PCR product (bp)**	**Reference**
**Universal primers for hemotropic mycoplasma species**	HBT-F HBT-R	5'-ATACGGCCCATATTCCTACG-3' 5'-TGCTCCACCACTTGTTCA-3'	595 bp	8
***Mycoplasma*** ***haemofelis*** ***Candidatus*** ***Mycoplasma* haemominutum**	Jns-F Jns-R	5'-ACGAAAGTCTGATGGAGCAATA-3' 5'-ACGCCCAATAAATCCG (A/G) ATAAT-3'	170 bp 193 bp	14
***Candidatus Mycoplasma*** ** turicensis**	Mt1-F Mt2-R	5'-GTA TCC TCCATC AGA CAG AA-3' 5'-CGC TCC ATA TTT AAT TCCAA-3'	488 bp	22
**GAPDH gene**	GAPDH-F GAPDH-R	5'-CCTTCATTGACCTCAACTACAT-3' 5'-CCAAAGTTGTCATGGATGACC-3'	277 bp	7

GAPDH: glyceraldehyde-3-phosphate dehydrogenase.

**Gene sequencing. **A 595 bp fragment of the 16S rRNA gene, using universal haemotropic mycoplasma primers; 5´-ATACGGCCCATATTCCTACG-3´ and 5´-TGCTCCACCAC TTGTTCA-3´ as forward and reverse primers designed by Criado-Fornelio *et al*. was amplified^22^ and positive products were subjected to sequencing process using the sanger technique (ABI, 96-capillary XL).^25^

**Statistical analysis. **Statistical analysis was performed using SPSS software (version 16.0; IBM, New York, USA). Evaluation of normal distribution of hematological data was performed by a 1-sample Kolmogorov–Smirnov test. Data were analyzed with Fisher’s exact test and the independent *t*-tests and *p* < 0.05 is considered statistically significant. Sensitivity and specificity tests were performed with chi-square test. Sequence Data analysis and phylo-genic tree construction were performed with Genious (version 6.1.5; Biomatters Ltd., Auckland, New Zealand 2013). The evolutionary history was inferred using the Neighbor-Joining method.^26^ The optimal tree with the sum of branch length = 0.74248083 is shown. The percentage of replicate trees in which the associated taxa clustered together in the bootstrap test (1000 replicates) is shown next to the branches.^26^ The tree is drawn to scale, with branch lengths in the same units as those of the evolutionary distances used to infer the phylogenetic tree. The evolutionary distances were computed using the Kimura 2-parameter method^27^ and are in the units of the number of base substitutions per site. The analysis was involved 62 nucleotide sequences. All ambiguous positions were removed for each sequence pair. Evolutionary analyses were conducted in MEGA (version 6.0; Biodesign Institute, Tempe, USA).^27^

## Results

From 185 samples, 47 (25.40%) were PCR-positive by universal haemotropic mycoplasma conventional PCR. The number of positive samples by species specific PCRs were *M. haemofelis* (n = 6), *Candidatus *M. haemominutum’ (n = 20), *Candidatus *M. turicensis’ (n = 5), *M. haemofelis *and ‘*Candidatus *M. turicensis’ (n = 2), and *M. haemofelis *and ‘*Candidatus *M. turicensis’ (n = 14). There were co-infections of different feline hemotropic mycoplasmas. 

From 47 hemoplasma positive samples, 10 (21.20%) had anemic hematological profiles (HCT < 24.00%) and 36 (76.50%) were male. All cats with only* M. haemofelis *infection or its co-infection with other species had anemic blood profiles. Overall anemia index factors including; hematocrit, red blood cells and hemoglobin in hemo-plasma positive samples are less than the same factors in hemoplasma negative cats. Data are shown in Tables 2, 3 and 4. Male cats were at higher risk of hemoplasma infection (*p* = 0.017, 95% confidence interval) with odds ratio of 2.699 greater than female infected cats.

**Fig. 1:**
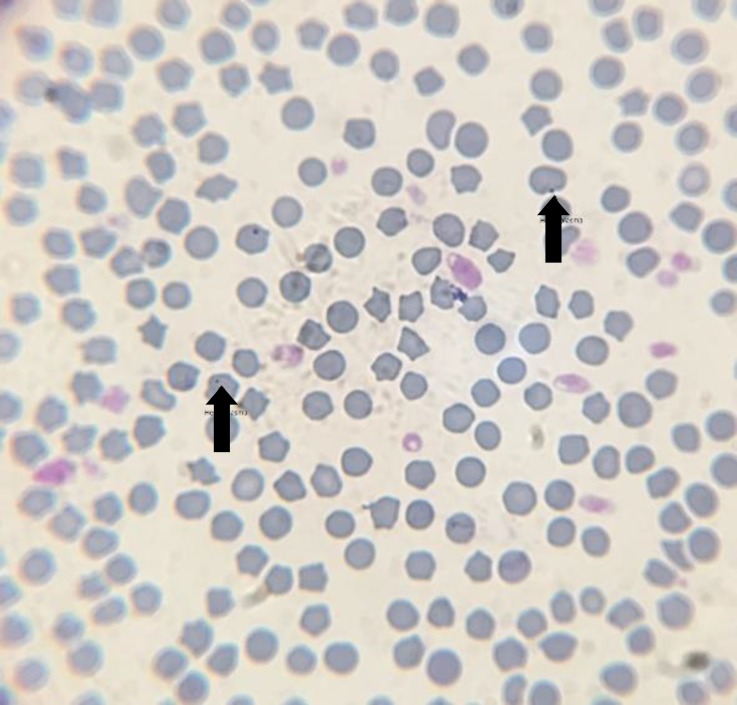
Wright-Giemsa stained cat blood smear at 100× with an oil immersion lens; hemoplasma bodies are shown with black arrows

Blood smears of 17 samples out of 185 total samples were positive for hemoplasmas (Fig. 1), of which five were negative with PCR. Using PCR as standard, cytology had a sensitivity of 28.57% and specificity of 96.50%.

The 16S rRNA gene sequences derived from this study were submitted to Genbank with accession numbers of KX253960, KX253961, KX253962, KX253963, KX253964 for 16S rRNA genes of ‘Ca. M. haemominutum’ KX253965, KX253966 for *M. haemofelis and *KX253967 for ‘*Ca*. M. turicensis’ from domestic cats.^28^

**Fig. 2:**
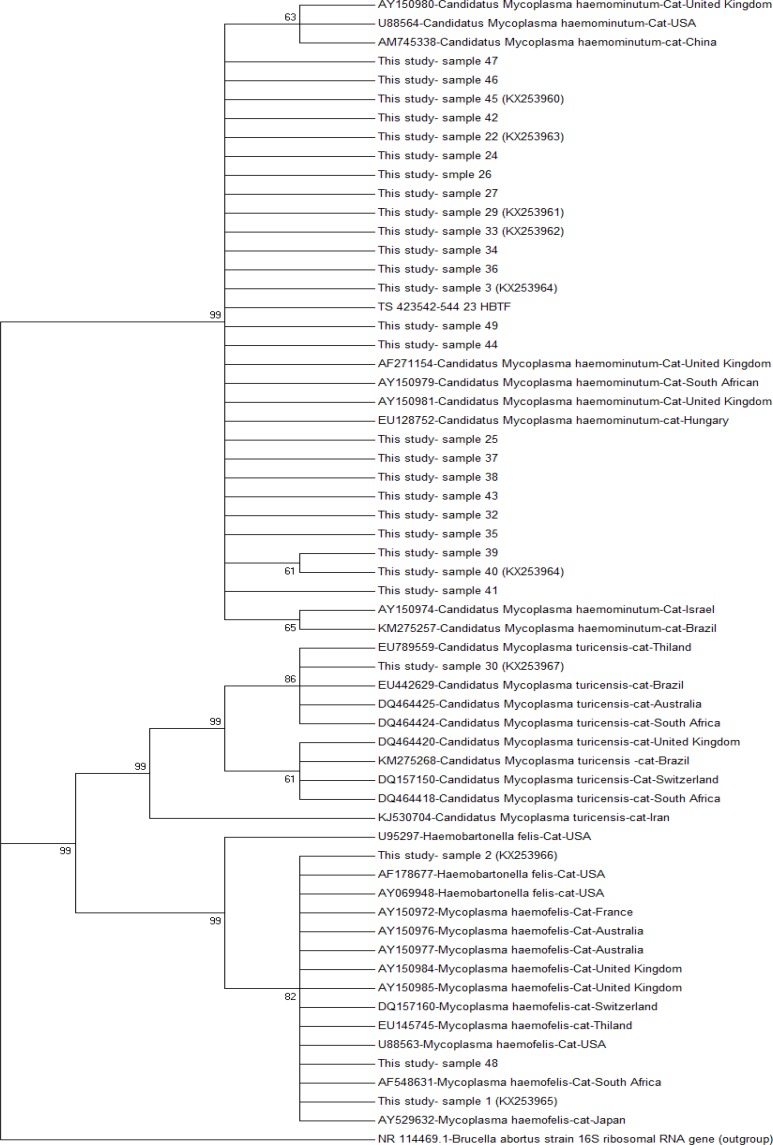
595 bp fragment 16S rRNA gene phylogenetic analysis; the hemoplasma species found in this study and domestic cat sequences existing in GenBank. The following sequences are shown; ‘*Ca. *M. haemominutum’ (cat, United Kingdom, AY150980; cat, USA, U88564; cat, China, AM745338; cat, United Kingdom, AF271154; cat, South Africa, AY150979; cat, United Kingdom, AY150981; cat, Hungary, EU128752; cat, Israel, AY150974; cat, Brazil, KM275257), ‘*Candidatus *M. turicensis’ (cat, Thailand, EU789559; cat, Brazil, EU442629; cat, Australia, DQ464425, cat, South Africa, DQ464424; cat, United Kingdom, DQ464420; cat, Brazil, KM275268; cat, Switzerland, DQ157150; cat, South Africa, DQ464418; cat, Iran, KJ530704), *M. haemofelis *(cat, USA, U95297; cat, USA, AF178677; cat, USA, AY069948; cat, France, AY150972; cat, Australia, AY150976; cat, Australia, AY150977; cat, United Kingdom, AY150948; cat, United Kingdom, AY150985; cat, Switzerland, DQ157160; cat, Thailand, EU145754; cat, USA, U88563; cat, South Africa, AF548631; cat, Japan, AY529632), *Brucella* abortus NR114469

Partial 16S rRNA gene sequence derived from the hemoplasma infected cats in the current study (accession number KX253960, KX253961, KX253962, KX253963, KX253964, KX253965, KX253966 and KX253967) showed high sequence identity to worldwide *M. haemofelis, *‘*Ca*. M. haemominutum’ and ‘*Ca*. M. turicensis’ sequences in Genbank.^7^^,^^14^^,^^28^^,^^29^ The KX253967 showed 97.16 to 100% sequence identity to ‘M. turicensis’ sequences. Sequences of *Ca*. M. haemominutum’ including KX253960, KX253961, KX253962, KX253963 and KX253964 ‘presented 94.12 to 100% identity to the reference sequence (accession NC 021007.1). *M. haemofelis *sequences derived from this study, KX253965 and KX253966, showed 98.82 to 99.28% sequence identity to reference *M. haemofelis *sequence (NR 103953.1), (Fig. 2).

**Table 2:** Sex distribution in hemoplasma PCR-positive and -negative cats

	**Male**	**Female**	**Total**
**Positive by smear examination**	11	6	17
**Negative by smear examination**	101	67	168
***Total***	112	73	185
**Positive by PCR**	36	11	47
**Negative by PCR**	76	62	138
***Total***	112	73	185

**Table 3:** Anemia distribution in three different hemoplasma species positive isolates

	**Mhf**	**CMhm**	**CMt**	**Mhf-Cmt**	**CMhm-CMt**	**Total**
**Anemic profile**	5	1	0	2	2	10
**Not-anemic profile**	0	15	5	0	17	37
**Total**	5	16	5	2	19	47

Mhf:* Mycoplsama haemofelis, *CMhm: *Candidatus *M. haemo-minutum, CMt: *Candidatus *M. turicensis, Mhf-Cmt: *M. haemofelis *and ‘*Candidatus *M. turicensis, CMhm-CMt:* M. haemofelis *and* Candidatus *M. turicensis.

**Table 4:** Hematological parameters of hemoplasma positive and negative cats *Data are presented as Mean ± SD

**Parameter**	**Hemoplasma positive **	**Hemoplasma negative **	**Reference range** ^30^	**Unit**
**Hematocrit**	25.40	35.16	29.00 - 45.00	%
**Hemoglobin**	8.80	13.20	8.00 - 14.00	g dL^-1^
**Red blood cells **	6.40	8.54	6.00 - 10.00	10^6^ µL^-1^
**Mean corpuscular volume**	50.00	47.60	41.00 - 54.00	fL
**Mean corpuscular hemoglobin**	16.10	15.32	13.30 - 17.50	pg
**Mean corpuscular hemoglobin concentration**	32.00	31.33	31.00 - 36.00	%
**Platelets**	1.80	3.70	2.30 - 6.80	10^5^ µL^-1^
**White blood cells**	6.80	16.80	5.50 - 19.50	10^3^ µL^-1^
**Segmented neutrophil**	2.13	9.56	2.50 - 12.50	10^3^ µL^-1^
**Band** **cell**	0.05	0.21	0.00 - 0.30	10^3^ µL^-1^
**Lymphocyte**	1.80	3.32	1.50 - 7.00	10^3^ µL^-1^
**Monocyte**	0.05	0.07	0.00 - 0.85	10^3^ µL^-1^
**Eosinophil**	0.04	0.20	0.00 - 1.50	10^3 ^µL^-1^
**Basophil**	0.00	0.00	Rare	10^3^ µL^-1^

* Age range of cats in this study was 3.34 ± 1.71 years old.

## Discussion

This study was performed on domestic cats in Iran to investigate the molecular aspects of feline hemotropic mycoplasmas. The presence and co-infection of known feline hemoplasmas were shown by our group in another study in 2014. Moreover, it has been shown that sex, age and fighting history are predisposing risk factors of hemoplasma infection in cats.^12^ In agreement with previous studies, data obtained from the current study confirm that sex is a risk factor for hemoplasma infection.^7^^,^^11^^,^^31^^,^^32^

 Anemia (HCT < 24.00%) was detected in all *M. haemofelis* positive cats, either the infection was solely by *M. haemofelis* or combined with other hemoplasma species (totally seven out of ten anemic-hemoplasma positive cats). Data are shown in Table 3. There are several reports that the most pathogenic feline hemoplasma species is *M. haemofelis.*^5^^,^^6^ Some studies described that retrovirus infections could worsen the severity of the hemoplasma-induced anemia either in *M. haemofelis* infection or in anaemia following infection with less pathogenic hemoplasmas such as ‘Ca. M. haemominutum’ and ‘*Ca.* M. turicensis’.^8^^,^^9^ Unfortunately, serologically or molecularly screenings of retroviral co-infections were not possible in this study, which prohibited us from knowing whether co-infection might result in the hematological abnormalities found specially in low pathogen hemoplasma-induced infection.

There was no anemic case, infected only by ‘*Ca.* M. turicensis’, but some co-infected cats with ‘*Ca.* M. turicensis’ exhibited an anemic hematological profile. This result is in agreement with other studies shown the low pathogenicity of ‘*Ca.* M. turicensis’ infection solely.^3^^, ^^33^^, ^^34^

Smear examination is not a sensitive diagnostic tool, which traditionally is applied primarily in diagnostic labs to detect hemoplasmas.^9^^,^^34^ Comparing hemoplasma screening PCR results, as a described standard for hemo-tropic mycoplasmas detection, smear results in the current study confirm the same outcome with a sensitivity of 28.57% and specificity of 96.50% for cytology examination.

Co-infection of different feline hemoplasma species has been described in previous studies. In a study by Aquino *et al*., coinfection of two or three feline hemoplasma species was reported. *M. haemofelis *and ‘*Ca*. M. haemominutum’ infection was the most frequent co-infection in the referred study. Meanwhile, ‘*Ca*. M. turicensis’ and ‘*Ca*. M. haemominutum’ co-infection was observed in the current study.^35^ In another study by Willi *et al*. in Switzerland, the association between ‘*Ca*. M. turicensis’ and ‘*Ca*. M. haemominutum’ has been shown which is in agreement with the results of present study. From 21 ‘*Ca*. M. turicensis’ positive samples, 14 samples (66.60%) were also ‘*Ca*. M. haemominutum’ positive.^7^

High sequence identity was observed between* M. haemofelis, *‘*Ca*. M. haemominutum’ and ‘*Ca*. M. turicensis’ isolates in this study and domestic cat derived sequences of three feline hemoplasmas in Genbank with no obvious geographical or host specificity grouping. Sequencing alignment with sequences derived from previous studies showed that worldwide isolated hemoplasmas are nearly identical irrespective of geographical or host origin.^2^^,^^36^^,^^37^

In uncultivable organisms such as hemoplasmas, phylogenetic analysis provides great information about their taxonomy. Several studies were investigated the phylogeny of hemoplasma on the basis of mainly two genes; 16S rRNA and RNase P RNA gene (rnpB) sequences.^2^^,^^29^^,^^36^^-^^38^

However, few studies have performed phylogenetic analysis on non-16S rRNA genes e.g., comparing the 16S rRNA gene, rnbp gene sequences have a higher nucleotide variation in closely related taxa.^28^^,^^37^^-^^39^

All hemoplasma isolates in this study were grouped within a single clade using 16S rRNA gene phylogenetic tree and were additionally subdivided into two groups; haemofelis group including two feline hemoplasma species, *M. haemofelis *and ‘*Ca*. M. turicensis’ and haemominutum group including ‘*Ca*. M. haemominutum’.

Hemoplasmas are not aggressive microorganisms with acute disease feature but could potentially cause anemia or deteriorate other infections like FeLV or FIV which could result in fatal anemia.^12^^,^^16^ To prevent clinical, diagnostic and therapeutic complications in pet clinics and having a greater health monitoring in cat populations, detection of subclinical and chronic infections like feline hemoplasmas could be very helpful.

Moreover, it should be considered that evolution relatedness and identity of these species in felids are so high and conserved with no obvious geographical or host specificity. 
